# Added-Value Compounds in Cork By-Products: Methods for Extraction, Identification, and Quantification of Compounds with Pharmaceutical and Cosmetic Interest

**DOI:** 10.3390/molecules28083465

**Published:** 2023-04-14

**Authors:** Carolina Morais Carriço, Maria Elizabeth Tiritan, Honorina Cidade, Carlos Afonso, Joana Rocha e Silva, Isabel F. Almeida

**Affiliations:** 1 Laboratory of Organic and Pharmaceutical Chemistry, Department of Chemical Sciences, Faculty of Pharmacy, University of Porto, 4050-313 Porto, Portugal; 2 CIIMAR—Interdisciplinary Centre of Marine and Environmental Research, Terminal de Cruzeiros do Porto de Leixões, Avenida General Norton de Matos, S/N, 4450-208 Matosinhos, Portugal; 3 Dimas & Silva, Lda. Industry, Rua Central de Goda 345, 4535-167 Mozelos, Portugal; 4 UCIBIO—Applied Molecular Biosciences Unit, MedTech, Laboratory of Pharmaceutical Technology, Department of Drug Sciences, Faculty of Pharmacy, University of Porto, 4050-313 Porto, Portugal; 5 Associate Laboratory i4HB—Institute for Health and Bioeconomy, Faculty of Pharmacy, University of Porto, 4050-313 Porto, Portugal

**Keywords:** *Quercus suber*, sustainability, cork by-products, extraction, isolation, quantification

## Abstract

The growing threat of climatic crisis and fossil fuel extinction has caused a boom in sustainability trends. Consumer demand for so-called eco-friendly products has been steadily increasing, built upon the foundation of environmental protection and safeguarding for future generations. A natural product that has been used for centuries is cork, resulting from the outer bark of *Quercus suber* L. Currently, its major application is the production of cork stoppers for the wine industry, a process that, although considered sustainable, generates by-products in the form of cork powder, cork granulates, or waste such as black condensate, among others. These residues possess constituents of interest for the cosmetic and pharmaceutical industries, as they exhibit relevant bioactivities, such as anti-inflammatory, antimicrobial, and antioxidant. This interesting potential brings forth the need to develop methods for their extraction, isolation, identification, and quantification. The aim of this work is to describe the potential of cork by-products for the cosmetic and pharmaceutical industry and to assemble the available extraction, isolation, and analytical methods applied to cork by-products, as well the biological assays. To our knowledge, this compilation has never been done, and it opens new avenues for the development of new applications for cork by-products.

## 1. Introduction

The cork oak, named by Linnaeus as *Quercus suber* L., is a slow-growing, evergreen, broad-leaved tree, belonging to the *Fagaceae* family. It tends to present sclerophyllous adaptivity, since it can tolerate, to a certain extent, periods of dryness and heat. Due to these characteristics, along with the preference for warm humid, and sub-humid conditions, this species’ geographical range of distribution is mainly concentrated around the western Mediterranean Basin (Portugal, Spain, Southern France, southern Italy, North Africa) and China [1,2,3]. Cork oaks show high plasticity and the capability to adapt to the challenging environmental conditions of these regions, as they are able to resist hot summers without rain and poorly fertile soils. More specifically, they prefer an annual mean temperature of 13–18 °C, not tolerating temperatures below −10 °C, and the combination of low rainfall and severe humidity, being able to grow in soils that present pH values between 4.8 and 7.0. The primary constraints to the survival and thriving of the cork oak are alkaline soils, water logging, and long frosts. However, in ideal conditions, it can live up to 200 years and reach a height of 25 m [1,2,3,4].

The cork oak landscapes, known as *montados* in Portugal, are a notable example of a well-balanced relationship between conservation and development for the welfare of both the people and the environment. Besides the fact that these forests constitute a very significant source of income in economically disadvantaged areas, they also represent environmentally sustainable systems, as the cork oaks ensure a significant retention of carbon in these habitats, perform an essential role in the regulation of the hydrological cycle of the *montado*, and, by promoting soil conservation and protection, also take part in the fight against soil desertification [5,6,7,8].

Unlike other species of evergreen oaks, *Q. suber* L. has the ability to grow a thick, homogeneous outer bark, with an outer layer constituted by suberized cells, which forms an impermeable, elastic, and homogeneous tissue, commonly known as cork [9,10]. This outer bark can be stripped from the tree, without the necessity for damaging or felling it and it has the ability to regenerate it, owing to the activity of a set of cells known in botany as the phellogen. The phellogenic tissue of the tree remains active throughout its lifetime, which is the reason for the cork’s homogeneity [4,9,11].

Cork has been used by mankind since remote antiquity, mostly for the construction of floating devices and plugs for liquid-containing reservoirs [1,12]. This last application was revived in the 17th century by famous monk Pierre Pérignon, who suggested using a cone-shaped cork piece to close bottles of Champagne, revolutionizing the use of cork [10]. Since then, the cork stopper has been the central choice by the wine industry for the protection of various beverages, and this has been the main application for cork extracted from *Q. suber* L. However, due to cork’s many interesting characteristics, such as low density, imperviousness to liquids, good thermal and acoustic isolation, high elasticity, and resistance to abrasion [1,4,10,13], it is also used for many industrial applications, such as the production of insulation corkboard and cork agglomerates for the building industry, as well as for decorative and general uses, and even the manufacture of transport and aerospace applications [14,15,16,17]. The cork sector is considered self-sufficient in its resources, due to the low carbon emissions associated with the extraction and transformation processes, as well as the practice of a concept known as circular economy, through the reutilization of a considerable amount of the production residues, and the procedures involving the harvesting and processing of cork are overall environmentally friendly [11]. Nevertheless, the production of most cork products generates significant waste, and it is estimated that, annually, 68 to 85 thousand tons of cork waste is generated by cork industries worldwide [18].

Currently, a key focus in all areas of research and development is reaching sustainability. The United Nations defines sustainable development as “development that meets the needs of the present without compromising the ability of the future generations to meet their own needs” [19]. In order to meet these goals, it is necessary to characterize and reutilize natural waste sources, especially as they can be important sources of bioactive compounds. The cork industry generates many by-products, some of which contain in their composition tannins, phenolic acids, terpenoids, and other compound classes with bioactive potential, which can be explored by other industries, namely the pharmaceutical and cosmetic industries [20,21]. In order to achieve this, it is first necessary to develop the most suited methods for the extraction, identification and quantification of these compounds. Additionally, it is also of great interest to study the potential biological activity of these substances, namely their therapeutic and cosmetic value. This work aims to compile and summarize the current knowledge on the methods that are used on the extraction, identification, and quantification, as well as biological characterization, of the added-value compounds of cork, more specifically those with pharmaceutical and cosmetic interest.

## 2. Cork—Chemical Properties

The properties of natural cork, such as the ones already mentioned in this work (low density, imperviousness to liquids, and thermal isolation), but also its chemical and biological inertia, are directly related to attributes such as its cellular and chemical composition. Regarding cell characteristics, cork cells are of small dimensions, have very thin walls, with no cell content, and become impermeable in the process of suberin deposition [10,12].

The chemical composition of cork, and how it differs from other tissues such as wood, is partly responsible for the role it plays in the protection of the living, active tissues of the trees from external aggressions [1]. This chemical composition has been extensively studied [10,22,23,24,25], and the relative abundance of its components is extremely variable and dependent on factors such as geographical origin, genetic origin, climate, and soil conditions, among others [26,27]. However, cork is typically constituted by suberin (≈40%), lignin (≈25%), polysaccharides (≈20%), and an extractive fraction, composed of lipidic and phenolic substances (≈15%). A low percentage, typically under 1%, is constituted by inorganic compounds.

### 2.1. Suberin

**Figure 1:**
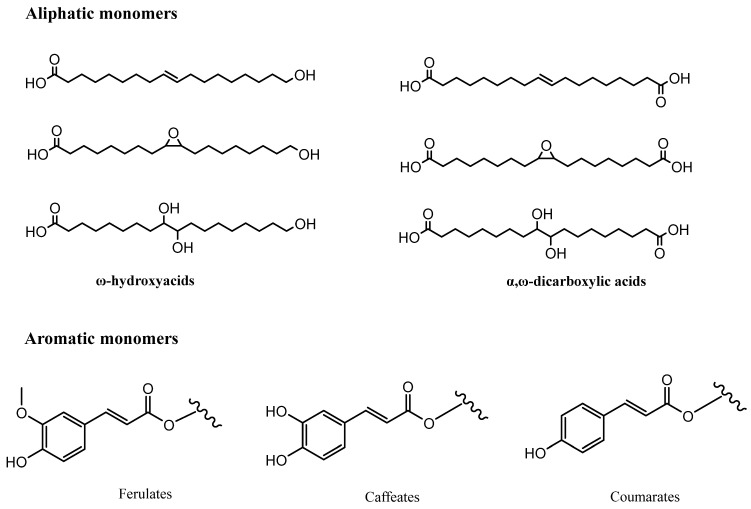
Structural formula of common monomers in suberin composition.

Suberin, the major component of the cork cell wall, is a lipophilic macromolecule, ubiquitous across the plant kingdom [28] and responsible for the impermeability of cork [1,29,30]. Suberose tissue is composed of an aliphatic domain as well as an aromatic one. The aliphatic domain consists of a polyester structure, made up of long-chain ω-hydroxyfatty acids and α,ω-dicarboxylic acids, linked by ester groups, and cross-linked by glycerol units. As for the aromatic domain, it is mostly constituted by hydroxycinnamic acid units esterified with glycerol or with ω-hydroxyfatty acids, thus bridging the two domains together (Figure 1) [28,31,32,33,34,35]. The major hydroxycinnamates present are ferulic acid derivatives and caffeates, as well as some coumaric acids, to a smaller extent [36,37].

Suberin is an important source of ω-hydroxyfatty acids, α,ω-dicarboxylic acids, and homologous mid-chain dihydroxy or epoxy derivatives; apart from suberin, these compounds tend to be scarce in nature, and they present an opportunity for the production of new cork-derivate materials [20,38]. Potential applications involve using suberin monomers for the preparation of ink additives, polyurethane and polyester materials [39,40], but also for the preparation of cosmetic products [41], especially when taking into consideration the potential antibacterial activity of the fatty acids present in suberin [42]. In fact, this property has already been exploited. Tamm et al. [43] established that suberin fatty acids from the bark of the birch tree (*Betula* spp.) could be incorporated into nanofibers for wound therapy as a synergistic antimicrobial biomaterial. More recently, Liakos et al. [44] have attempted to use suberin as a vehicle for drug delivery, through the creation of suberin nanoparticles incorporating *trans*-cinnamaldehyde and loaded with paclitaxel, which demonstrated some antitumor activity against HepG2 (human hepatocellular carcinoma) cell lines. Additionally, suberin extracts have also proven to have a protective effect on induced mutagenicity [45]. In this regard, and despite natural cork being richer in suberin than cork powder [46], this and other cork by-products can be important sources of suberin. In fact, Gandini et al. [38] estimated that cork powder could be the source for 16,000 tons of suberin per year. Virgin cork too, which is not used for cork stopper production, present a higher amount of suberin than reproduction cork, which makes it an attractive source of antimicrobial and antimutagenic compounds.

### 2.2. Lignin

**Figure 2:**
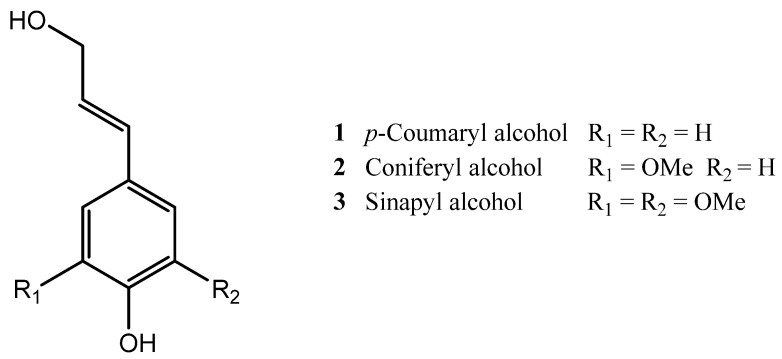
The three phenylpropanoid building blocks of lignin.

Suberin is linked to lignin, the structural phenolic polymer of the cork cell wall which provides it with mechanical support and rigidity. Lignin is assembled, by enzymatic polymerization, from three phenylpropane monomers: *p*-coumaryl alcohol (**1**), coniferyl alcohol (**2**) and sinapyl alcohol (**3**) (Figure 2). This characterization of lignin was first elucidated by the works of Marques et al. [47,48] through permanganate oxidation and thioacidolysis. Lignin presents a branched macromolecular structure, through the covalent linkage of the phenylpropanoid units; they can also exhibit different functional groups, such as aromatic and aliphatic hydroxyls, benzyl alcohol, ether, carbonyl and methoxy groups [1,49]. Similar to suberin, lignin is a hydrophobic polymer, which is also covalently bound to polysaccharide units, filling the gaps between cellulose and hemicellulose and creating a lignin-cellulose matrix that connects with the aromatic domain of suberin [4,50].

Recently, lignin was defined as an “important natural bioresource” for the production of biofuels and the obtention of added-value chemicals [51]. Indeed, lignin presents interesting properties, such as being a natural broad-spectrum sun blocker (due to the presence of functional groups such as phenolic units, ketones and other chromophores, as well as intramolecular hydrogen bonds). Besides these ultraviolet (UV) radiation absorption properties, the phenolic groups also have free radical scavenging ability, which confer antioxidant capacities to lignin [52,53]. These two attributes result in a potential exploitation of lignin for incorporation into high-end products—namely, sunscreens, making them more sustainable and natural products, both of which are appealing characteristics for current consumers. In regard to cork by-products, cork powder is an interesting source of lignin, as it presents a higher amount of this substance than natural cork [46].

### 2.3. Polysaccharides

Apart from suberin and lignin, cork cell walls are also composed by polysaccharides, the main ones being cellulose and hemicellulose. The extraction of polysaccharides is typically achieved through acid, basic or enzymatic hydrolysis, followed by solvent extraction [4]. After the identification of cellulose and hemicellulose in cork [54,55], Pereira & Tomé [22] determined the monosaccharide composition of virgin and reproduction cork after acid hydrolysis of glycoside linkages and verified that, in both types, the main monosaccharides were glucose and xylose. Together with lignin, these polysaccharides contribute to the cell’s structural support and rigidity.

### 2.4. Extractives

Finally, a fraction of cork cell wall components that raises interest for its bioactive properties is the “extractives” fraction, so-called due to the means of separation and attainment of the compounds that constitute it—that is, simple solvent extraction. The reason why these compounds are easily removed through extraction is because they are not chemically bound to other structures, similar to the ligno-cellulosic matrix which is, itself, linked to suberin. Even though they tend to be ubiquitous in plants, cork possesses a higher number of extractives than regular wood, although the amount and type of extractives present are affected by factors such as geographical and genetic origin of the tree, its age and the soil and climate conditions [1,56].

**Figure 3:**
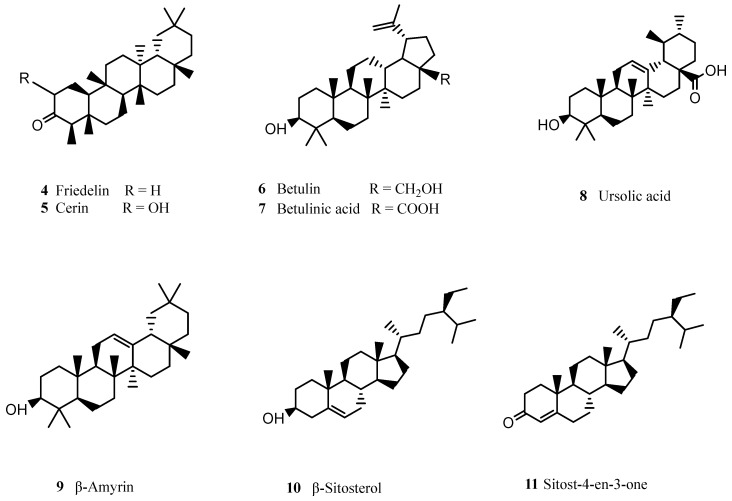
Chemical structures of the main triterpenoids identified in cork.

The two major groups of compounds present in the “extractives” fraction are waxes and phenolic compounds. Regarding the chemical characterization of each of these two groups—waxes and phenols—waxes incorporate a variety of compounds, such as triterpenoids, fatty acids, n-alkanes (C16–C34) and n-alkanols (C20–C26) [1,57]. Concerning the triterpenoid fraction, it includes friedelin (**4**), cerin (**5**), betulin (**6**), betulinic acid (**7**), ursolic acid (**8**), β-amyrin (**9**), β-sitosterol (**10**), sitost-4-en-3-one (**11**), and derivatives of lupan and friedelan (Figure 3) [1,57,58,59]. Triterpenes appear to be present in esterified forms in natural cork, as demonstrated by Touati et al. [57], who reported a significant increase in the amount of triterpenes after alkaline hydrolysis: the amount of triterpenes per kg of dry weight more than tripled. Several triterpenes possess biological activity: betulinic acid (**8**), for instance, exhibits antitumor activity against many types of cancer (such as breast and lung cancer), but also anti-inflammatory and anti-diabetic activity [60]; friedelin (**4**), has demonstrated bioactive potential as an antiulcerogenic [61,62], as well as hypolipidemic [63] agent.

As for the fatty acids present in this fraction, they are both saturated and unsaturated, with hexadecenoic and docosanoic acids being the predominant ones, followed by 9,12-octadecadienoic, *cis*-9-octadecenoic and *trans*-9-octadecenoic acids [57]. After alkaline hydrolysis, it was also possible to identify 9,12,15-octadecatrienoic acid and a hydroxyacid (22-hydroxydocosanoic acid). The beneficial health effects associated with fatty acids, particularly ω-3 and ω-6 unsaturated fatty acids, are now common knowledge among popular culture, particularly for their role in the prevention of cardiovascular diseases, certain types of cancer, and type 2 diabetes [64]. Thus, exploring the potential of cork, and more specifically cork by-products, as sources of these compounds, would be an interesting way of raising their economic value.

**Figure 4:**
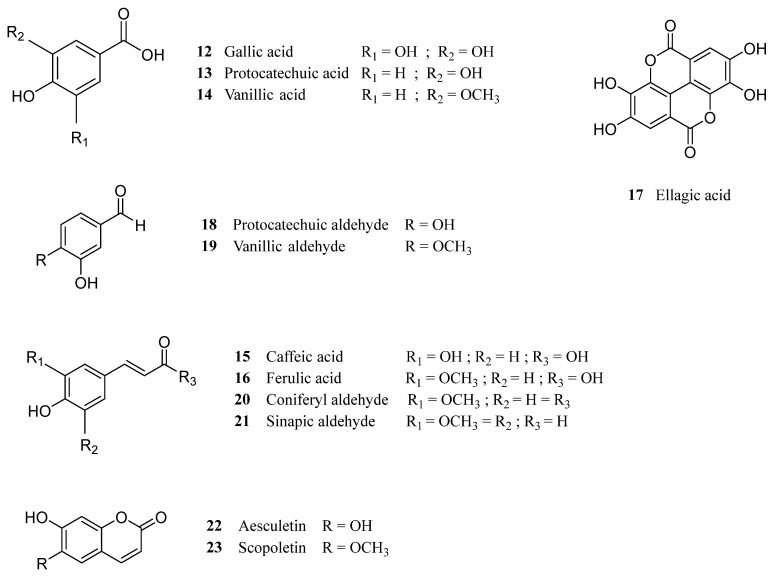
Chemical structure of the main phenolic acids, aldehydes and coumarins identified in the “extractive” fraction of cork.

Much like the aliphatic portion of the extractives, the phenolic compounds present in cork also exhibit important bioactive properties and potential use in human health, reason for which the chemical composition of this fraction has been extensively studied [23,57,65,66]. This fraction comprises simple phenols, also referred to as low molecular weight polyphenols (LMWP), and polymeric phenols, tanning being the most significant ones. Regarding the LMWP in cork, many studies have reported the identification of gallic (**12**), protocatechuic (**13**), vanillic (**14**), caffeic (**15**), ferulic (**16**) and ellagic (**17**) acids, as well as protocatechuic (**18**), vanillic (**19**), coniferyl (**20**) and sinapic (**21**) aldehydes, but also coumarins such as aesculetin (**22**) and scopoletin (**23**) (Figure 4) [23,65,66,67]. Many of these compounds present promising biological activity: ferulic (**16**) and protocatechuic (**13**) acids, for instance, have exhibited anti-inflammatory, antioxidant, anticancer and antioxidant properties [68,69]. Gallic acid (**12**) has also been proposed for the formulation of cosmetic products, due to its antioxidant, antimicrobial, and anti-inflammatory potential [70].

**Figure 5:**
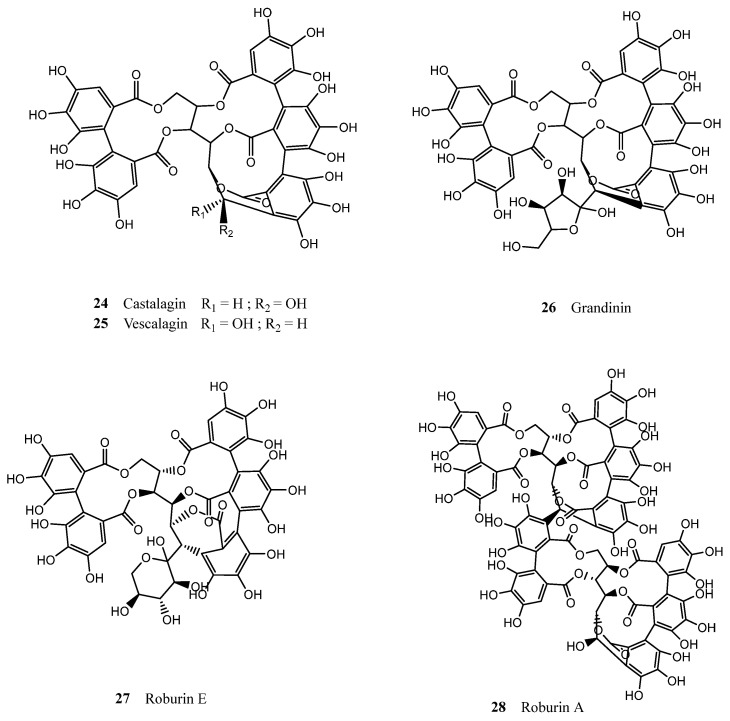
Chemical structures of the main tannins present in cork.

As for the tannic composition of cork, it seems to be dominated by hydrolysable tannins, in particular ellagitannins. The major tannins found in natural cork are two diastereoisomers—castalagin (**24**) and vescalagin (**25**), followed by grandinin (**26**) and roburin E (**27**) and A (**28**), in decreasing order of abundance (Figure 5) [23,65,71]. The pharmacological properties associated with tannins are diverse, ranging from antimicrobial, to antiparasitic, anti-inflammatory, antiviral, and even antiapoptotic [72]. In the genus *Quercus*, in particular, this family of compounds has exhibited antioxidant [73,74] and antidiabetic [75] properties.

## 3. Cork Processing

### 3.1. Cork Harvesting and Industrial Transformation

Cork is first harvested from the cork oak when its trunk grows to a perimeter of about 70 cm, which happens when the tree reaches 20 to 30 years of age [12]. The following extractions occur periodically, at 9- to 12-year intervals, preferably in late spring or summer, when the phellogen is in full meristematic activity, so the removal of cork from the stem and branches does not damage the tree [76]. In each harvest, it is possible to extract 40 to 60 kg of cork per tree [77].

The life cycle of the cork oak generates three types of suberose tissue: the first extracted material, known as virgin cork, presents irregular structure, thickness and density—thus, due to its many morphological defects, it is only used for the production of agglomerates; the second extraction, which generates the so-called “first reproduction cork”, still lacks sufficient quality for cork stoppers; only the second reproduction cork, corresponding to the third and following extractions, possesses the necessary qualities for the production of natural wine stoppers [1,4,56].

Post-harvest, cork planks are deposited on a dry field, and then transported into a yard, for chemical and structural stabilization. Once they have reached an adequate level of dampness, the planks are boiled in water for an hour, a process which has the purpose of expanding the planks, to increase their thickness and, therefore, lower the material’s density, as well as increase its smoothness and elasticity. The boiling also succeeds in cleaning the cork and in extracting some hydrossoluble substances from it. The boiling operation is followed by another period of stabilization and ventilation, after which the cork planks are separated according to thickness, porosity and appearance—the best planks are then selected for stopper production, and the ones rejected are subjected to grinding, and reutilized for the production of agglomerates. Portugal is the world’s leading cork producer, with an annual production of about 85,000 tons, having France as the main client [78].

### 3.2. Sub-Products and Added-Value Compounds

Each of the stages previously described generates by-products—cork materials that do not possess the requirements to be transformed into cork stoppers. This, however, does not mean that these residues cannot be utilized for other purposes and applications, in fact, the characterization of these by-products and their valorization is becoming increasingly more important, with the emerging exhaustion of fossil fuels and the ever-growing need for alternative, renewable sources for the obtention of energy and materials.

One of the main cork by-products are cork granulates, which arise from the granulation, or grinding, of scraps and parings of virgin cork and cork materials that do not pass quality tests, thus resulting from many stages of cork stopper production. Despite having some applications—they are used for thermal and acoustic insulation—their main utilization is for the obtention of agglomerates [11]. However, cork granulates present an interesting chemical composition, with high amounts of extractives, thus constituting a source of bioactive compounds. These include phenolic constituents, such as ellagic (**17**), gallic (**12**), protocatechuic (**13**), caffeic (**15**), ferulic (**16**) and coumaric acids, as well as some coumarins, such as aesculetin (**22**) and scopoletin (**23**), but also a considerable number of tannins, namely castalagin (**24**), tergallic-C-glucose, roburins (**27**, **28**), and vescavaloninic acid (**32**) [65,66,79,80].

Agglomerates, which are also known as black, pure or expanded agglomerates, are the result of the agglutination of the aforementioned cork granules through a process carried out by the autoclave, without the use of synthetic resins as binding agents, but rather by subjecting the granules to superheated steam. Their applications include thermal, anti-vibratic and acoustic insulation, mostly for the building industry, as they retain many of the properties of natural cork, namely its low conductivity of sound and temperature and high mechanical resistance [11,81]. Meanwhile, the production of agglomerate through the treatment of cork residues at high temperatures generates a residue known as black condensate, which consists in the product that arises from the vapors formed in the autoclave pipes during said treatment; black condensate is then systematically collected from the pipes and its combustion generates energy. Black condensate is extremely rich in extractives—more specifically in triterpenes, of which friedelin (**4**) is the most abundant, and in phenols, the predominant ones being ellagic acid (**17**), coniferaldehyde (**20**), aesculetin (**22**) and gallic acid (**12**) [80]. Black condensate may also constitute a source of ω-hydroxy fatty acids [82].

Cork granulates can also be subjected to agglutination with synthetic or natural resins, resulting in a product called composition cork, which can be used to produce agglomerated cork stoppers, floor coverings for construction, utilities for the automotive and aeronautical industries, among others [11,83]. An application of cork granules that did not entail the production of agglomerates, but instead took advantage of this by-product for the preparation of activated carbons, was achieved by Mestre et al. [84]; furthermore, these chemically activated carbons possessed the capacity to remove a number of pharmaceuticals residues from water, among which ibuprofen and paracetamol.

**Figure 6:**
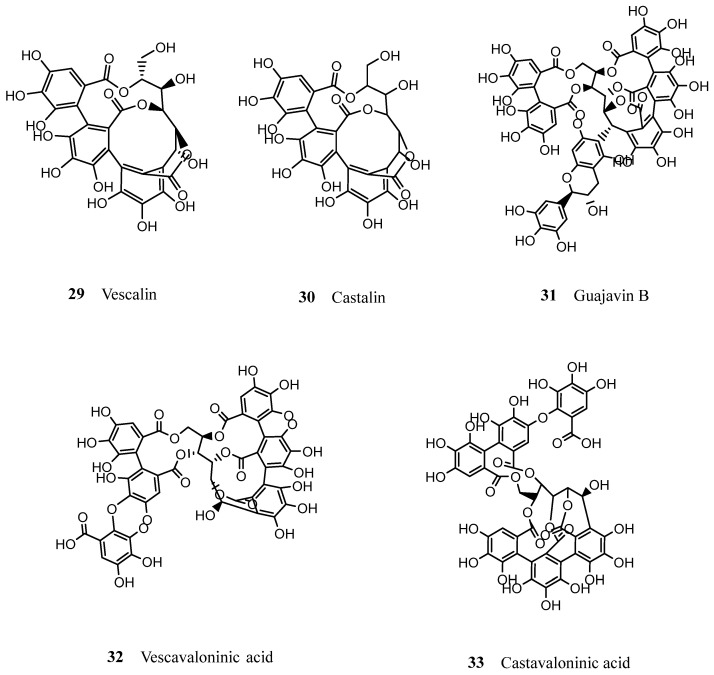
Chemical structures of recently identified tannins in cork by-products (cork powder and granulates).

All of the by-products described above have found new applications and possess economical value. However, not all of the residues that arise from the industrial processing of cork are adequate for the production of agglomerates, and smaller particles are segregated by granulometric separation. These cork particles constitute 25–30% of the raw material and are the major by-product produced from the cork industry—this sub-product is known as cork powder. Cork powder is produced in all stages of the industrial transformation of cork, including grinding, removal of impurities, cutting and sanding of planks, and even the production of other sub-products, such as agglomerates [85]. There are different types of cork powder, arising from different stages of cork processing, as proposed by Gil [85], and they differ slightly in terms of constitution and characteristics. However, the overall composition of cork powder is, from a chemical and pharmacological point of view, a very interesting one. Despite having lower amounts of suberin in comparison to natural cork, cork powder is richer in Klason lignin [46] which, as previously stated, could be further exploited and incorporated in the manufacture of many cosmetic products, such as sunscreens. In terms of extractive compounds, cork powder could also be a source of bioactive triterpenic and lipophilic substances. In a study aiming to identify the major waxes from industrial cork by-products, Sousa et al. [82] reported that the main components present in cork powder were betulinic acid (**7**), cerin (**5**) and friedelin (**4**). According to Santos et al. [80], the major phenolic compounds found in this sub-product were ellagic acid (**17**), gallic acid (**12**) and aesculetin (**22**). In relation to cork granulate, cork powder presents the same polyphenol composition, although in different quantities; according to Reis et al. [79], both of these by-products seem to present a small amount of complex tannins, such as acutissimin A and B, additionally to the ellagitannins already reported: castalagin (**24**), vescalagin (**25**), roburin A (**28**) and E (**27**) and grandinin (**26**). The same author also reported the identification of new tannins in these cork residues (cork powder and cork granulate), such as vescalin (**29**), castalin (**30**), guajavin B (**31**), vescavaloninic acid (**32**) and castavaloninic acid (**33**) (Figure 6). The main application of cork powder is serving as combustion fuel for the production of energy which contributes to the maintenance of the industry itself [86], but it also serves purpose in agriculture, in the building industry and, even in the cork industry itself, where it can be used as a filling agent to enhance the quality of cork stoppers [85]. Another interesting application for cork powder, which has been exploited commercially, is its use on biosorption for many kinds of pollutants (namely, insecticides and heavy metals) [87,88]. However, neither of these applications fully explore the potential of cork powder and its chemical richness, as they do not take advantage of this promising source of bioactive compounds, which can be incorporated into pharmaceuticals and cosmetic products.

Finally, a waste residue which also arises from the cork stopper industry, more specifically from the boiling of the cork planks, are the cooking wastewaters. These wastewaters constitute a major source of waste in the cork industry, generating a volume of 1500 L per ton of cork, despite the fact that it may be recycled twenty to thirty times [89,90]. These effluents present elevated toxicity, due to the extraction of certain compounds from cork during the immersion (such as pentachlorophenol, 2,4,6-trichloroanisol, benzoic acid, and some phenols), as well as the use of herbicides and pesticides in the montados, leading to their absorption by the oak tree, and their presence in the boiling wastewaters [91]. As such, the necessity for the valorization of this particular waste of the cork industry arises from two facts: its high phenolic content, and the fact that these waters tend to be discharged to the environment without previously being submitted to any kind of treatment—in spite of their low biodegradability and high toxicity. Therefore, on account of the methods for treating these wastewaters being sophisticated and expensive, the characterization of their chemical constituents becomes even more important, as a means of encouraging their valorizations and, hopefully, promoting the decrease of the pollution impact of this affluent. The chemical composition of these wastewaters is variable, as it depends on the composition of the cork itself, as well as the number of times it is boiled [89]. Minhalma & de Pinho [92] identified, through reverse phase high performance liquid chromatography (HPLC) method, the following phenolic solutes present in cork processing wastewaters: gallic acid (**12**), protocatechuic acid (**13**), syringic acid, ferulic acid (**16**), vanillic acid (**14**) and ellagic acid (**17**), which was, by far, the most abundant phenol in this residue. These phenols do not seem to be readily bioavailable, which is an impediment to the recovery of these compounds for utilization for new purposes [90].

In sum, there are many waste residues and by-products generated from the industrial manufacture of cork stoppers, due to the high-quality demands of the industry. Resulting cork materials that do not meet these demands are, however, still interesting from a pharmacological perspective, as most of them present a diverse and rich chemical composition. A summary of the most commonly attained compounds from each by-product is present in Table 1. Many of the compounds they possess can be recovered and exploited by various industries, such as the agricultural, food, cosmetic and pharmaceutical industries.

## 4. Extraction, Purification and Analytical Methods for Added-Value Compounds in Cork

### 4.1. Methods for Extraction

The very first step in the attainment of a natural compound of interest from the source material is extraction. There are two main types of extraction: solid/liquid and liquid/liquid extraction. These can be further divided into two categories: conventional techniques—which include maceration, percolation and Soxhlet extraction, steam distillation, infusion, and others—and modern techniques, such as ultrasound-assisted extraction (UAE), microwave-assisted extraction (MAE) and pressurized liquid extraction (PLE), for example. The conventional techniques, despite being the most widely used, present certain limitations, such as long extraction times, low selectivity, elevated costs due to the use of large volumes of organic solvents, and high energy demand, which are associated with harmful environmental effects, and the possibility of thermal degradation of labile compounds. Some of these drawbacks can be avoided by using modern extraction techniques, such as the aforementioned ones, as they grant higher selectivity, shorter extraction times and lower consumption of organic solvents. Alternatively, one can also use ‘greener’ solvents, such as water and supercritical fluids [93,94]. The choice of the method for extraction will rely on the properties of the target compounds, the water content of the starting material, and the aims of the overall procedure. As for the selection of the solvent, it should take into consideration certain aspects, such as its inertia or possibility of chemical interaction, its solubility, selectivity, cost and toxicity [95].

Concerning the bioactive compounds from cork by-products, a methodology often applied is a specific type of solid-liquid extraction—Soxhlet extraction [66,80,82]. The extraction with the Soxhlet apparatus is useful when the target compound has limited solubility in a certain solvent, while the undesired compounds are insoluble [96]. However, it presents certain disadvantages, namely the need for a long time period of extraction and considerable volume of solvent. Additionally, the use of a solvent with a high boiling point will implicate the use of a high temperature, which could result in the thermal degradation of the target compounds, if they are thermolabile, as well as a high energy consumption [97].

In extraction procedures, the choice of the extracting solvent will determine what type of components will be extracted—this is also true in regard to cork by-products: when using polar solvents, such as methanol, ethanol and water, the compounds extracted will be of phenolic nature; however, when using solvents of low polarity, such as DCM [57,66,80,82] and chloroform [79], the compounds that will be extracted will be mostly of low polarity. Within the “extractive” fraction of cork, which consists in the portion of compounds more easily extractible, these low polarity compounds include the terpenoids, such as betulinic acid (**7**), friedelin (**4**), cerin (**5**), betulin (**6**), β-sitosterol (**10**) and others (Table 2). These lipophilic extractives can be obtained through Soxhlet extraction, as was demonstrated by Sousa et al. [82], from two distinct by-products of the cork industry, cork powder and black condensate. As indicated in Table 2, the results suggest that black condensate would be a better source for these compounds, as it allowed for an overall better yield. The procedure consisted in a sequential Soxhlet extraction, involving three solvents, DCM, methanol and water, for 10 h with each one. In the case of cork powder, DCM managed to extract over 80% of the quantity of betulinic acid (**7**), cerin (**5**), friedelin (**4**), betulin (**6**) and β-sitosterol (**10**) that were present in the total extract (DCM, methanol and water), making it unnecessary to perform the full sequential extraction with three solvents if the goal is to extract one of these components. However, other compounds, such as lupeol and ursolic acid (**8**), were only extracted when using methanol and water. In regard to the black condensate, the DCM extracts were also submitted to alkaline hydrolysis with sodium hydroxide, which promoted the cleavage of existing ester bonds, and thus it was also reported the extraction of considerable amounts of aliphatic alcohols, fatty acids, α,ω-alkanedioic acids and interesting phenolic compounds, such as ferulic acid (**16**), vanilylpropanoic acid, benzoic acid, and other compounds, present in smaller quantities. Touati et al. [57] have also described a similar procedure of extraction and hydrolysis conditions, differing only in a shorter extraction time (6 instead of 10 h), giving rise to the identification of a much larger diversity of compounds. In fact, besides triterpenoids, Touati et al. also managed to identify a variety of fatty acids, diacids, hydroxyacids and long chain aliphatic alcohols in the DCM extract submitted to alkaline hydrolysis with sodium hydroxide [57].

In other circumstances, when the goal was to extract compounds of high polarity, such as phenolic acids, aldehydes, and tannins present in the “extractives” fraction of cork by-products, the Soxhlet extraction with DCM, chloroform or other low polarity solvents serves a different purpose, which is of sample pretreatment. This stage ensures the removal of more lipophilic components, before the extraction of the desired compounds, as was performed by Santos et al. [66,80]. In both works, the Soxhlet extraction with DCM was simply the preceding process for the extraction of the compounds of interest, phenolic compounds. The extraction of phenolic compounds from cork by-products, such as cork granulate and black condensate, is commonly achieved through the suspension of the resulting residues from the DCM extraction in a solvent of high polarity. This solvent may be a mixture of methanol and water, in different proportions (80:20 or 50:50, *v*/*v*), or simply methanol, both have the capacity to extract highly polar compounds [66,67,80]. Santos et al. [66] attempted two different extraction procedures for the isolation of phenolic compounds in granulated cork. After the extraction of lipophilic constituents with DCM, the solid residue was submitted to two different methodologies. One of the fractions was suspended in methanol:water (80:20, *v*/*v*) for 24 h at room temperature and, after the removal of the methanol present in the suspension, the aqueous solution was extracted three times with diethyl ether, as previously proposed by Conde et al. [67]. A second fraction was, instead, subjected to extraction with methanol for 6 h, yielding one of the extracts. A third extract was obtained by reflux with water for 6 h after the methanol extraction previously mentioned. As compiled in Table 2, this last procedure resulted in a considerably higher extraction yield. The authors proposed that a substantial fraction of phenolic components was not extracted when using the methanol:water mixture as extracting solvent; however, these extraction conditions allowed for a higher yield of certain substances, namely ellagic and caffeic acids, and also led to the identification, for the first time in cork extractives, of salicylic acid, eriodictyol and naringenin, as well as trace amounts of quinic acid. A successful example of these extraction conditions (using methanol: water, 50:50, *v*/*v*, as solvent) was then reported by Santos et al. [80] and, later, by Touati et al. [57], who achieved much higher yields for all compounds except ellagic acid (**17**). A possible reason for the distinct extraction yields could be the lack of the extraction step with diethyl ether which was performed three times in the procedure described by Santos et al. It also benefitted in terms of the identification of new compounds, such as methyl gallate, brevifolin-carboxylic acid, chlorogenic acid, among others. More recently, Reis et al. [79] have studied the optimum conditions for the recovery of tannins from two cork by-products, cork granulate and cork powder. A similar sample pretreatment was performed for the removal of lipophilic components, using chloroform instead of DCM. In order to extract the tannins present in these by-products, the defatting process was then followed by maceration of the granulates and powder with an aqueous solution of acetone, using different proportions of each solvent, as well as different time and temperature conditions. The resulting supernatant was then subjected to precipitation with ethanol, in order to remove polymerized tannins, which were centrifuged, and extracted with ethyl acetate. The aqueous phase was then purified by solid-phase extraction, and the tannins were eluted with a very polar mixture consisting of methanol:acetone:water (3:1:1). Results suggest that cork powder is richer in tannins than cork granulate, as the yield was superior (in many cases, more than double) for all the compounds extracted from cork powder.

Another interesting example was the one performed by Fernandes et al. [65,98], who selected a wine-model solution, consisting of 12% ethanol and 5 g/L tartaric acid buffered to pH 3.2, for the extraction of phenolic compounds from cork granulate. After the removal of the ethanol, the aqueous residue was spray dried and submitted to extraction with ethyl acetate (three times). Finally, the compounds were fractionated, through a procedure which will be described below (Section 4.2).

Non-conventional methods of extraction have also been applied to cork. For instance, Castola et al. [58] attempted supercritical fluid extraction (SFE) on cork granulates. Due to the distinct properties supercritical fluids present, such as a liquid-like density, high diffusivity, low viscosity and good solvating power, they exhibit high penetrating power, which is why they have been employed for the extraction of natural products since the end of the 1970s [99]. The most frequently used solvent for SFE is carbon dioxide (CO_2_), for being generally recognized as safe, environmentally innocuous and presenting low cost. Additionally, it is easy to recover after extraction, providing solvent-free analytes, and it can be utilized at low temperatures, as it possesses a moderate critical temperature, thus avoiding the degradation of thermally sensitive components [100]. For these reasons, supercritical CO_2_ was the selected solvent for SFE by Castola et al., who established a comparison between this method of extraction to a conventional Soxhlet extraction using DCM as extracting solvent [58]. SFE allowed for a slightly better yield of triterpene compounds from cork granulate, while requiring less time, and avoiding the use of solvents that endanger the environment. However, due to its low polarity, CO_2_ is not as successful for the extraction of high polarity components, such as the phenolic acids present in cork by-products which are so interesting from a pharmacological point of view. A way of overcoming this limitation is through the use of a modifier (co-solvent) which can promote changes to the solvent properties of supercritical CO_2_ [100]. Overall, SFE presents many advantages over conventional extraction techniques, the main ones being the high efficiency rates, associated with lower extraction time periods and the non-use of organic solvents, thus constituting a greener alternative for the extraction of natural products, while still being a versatile technique.

Another non-conventional method of extraction was carried out by Cunha et al. [51]. In this work, cork granulates were subjected to subcritical water extraction (SWE), with different temperature values (120, 150 and 200 °C), aiming to obtain extracts enriched in different compounds. It was possible to extract approximately 96% of the total phenolic content present in the granulates, and all of the extracts demonstrated antioxidant capacity. SWE is a promising technique for the extraction of bioactive compounds from plants, in particular compounds with antioxidant potential. Water at subcritical state presents higher solvating power and diffusivity and a decrease in viscosity and polarity; extraction with subcritical water takes less time and less volume of solvent, as well as allowing for the recovery of a more concentrated extract [101]. Additionally, a microwave-assisted extraction approach was attempted by Bouras et al. [102], with the aim of extracting polyphenols with antioxidant activity from the bark of cork oak tree, using hydroalcoholic solutions with variable proportions of water, ethanol and methanol, as solvents. The efficiency of this method was demonstrated in the extraction of antioxidant phenolic and sinapic acids present in cork residues, as well as the fact that this is a greener approach to the recovery of these bioactive compounds, makes it an interesting technique for the extraction of these substances from cork by-products.

The dominance of conventional techniques over modern ones for the extraction of bioactive compounds from cork by-products is still very pronounced, as illustrated in Table 2. However, there is still a great need to develop new and greener extraction methodologies that will allow for a satisfactory recovery of bioactive compounds present in cork granulates and powder, black condensate and other by-products.

### 4.2. Methods for Purification

The product obtained from an extraction procedure is a quite complex mixture of compounds, that require further purification. This purification will be dependent on chemical or physical differences that the compound of interest possesses relatively to the general mixture resulting from the extraction. Chromatography is a widely used method for the purification of desired constituents from sample extracts, because of its simplicity, generally elevated separation efficiency and swiftness [103]. Among chromatographic techniques, a myriad of procedures is available to choose from, based on different separation principles—different adsorption properties, ionic strengths, dimensions, or affinity to an aqueous or organic solvent [104]. However, this type of fractionation is not commonly applied to cork by-product extracts. Within literature reports, as far as we known, only two research articles have reported the application of this kind of procedure to cork by-products. Fernandes et al., 2009 [65] described the fractionation of cork phenolics, namely tannins, from cork granulates by preparative HPLC, using water/acetic acid (98:2, *v*/*v*) and acetic acid/acetonitrile/water (2:20:78, *v*/*v*/*v*) as mobile phase. This was a suitable technique for the attainment of an acceptable quantity of pure compounds in an easy and economical way; however, as the authors did not quantify the isolated tannins, it is not possible to state that it enabled a satisfying yield for this particular case. Later, the same authors [98] reported the fractionation of phenolic compounds resorting to column chromatography, a method previously described for the fractionation of oligomeric and polymeric procyanidins from grape seeds [105]. Using methanol as mobile phase, various fractions, composed of low molecular weight phenolics—such as gallic (**12**), caffeic (**15**) and ferulic acid (**16**)–tannins—such as ellagitannins (**29**, **39**) as well as complex tannins, and others were separated on the basis of their polarity.

### 4.3. Analytical Methods for Identification and Quantification

In past years, the separation of target compounds from an extract or matrix and their identification and quantification were entirely separate processes. The analytical chemist would separate the substances of interest through methods such as crystallization, filtration and chromatography, and only afterwards the compounds would be identified. Methods of quantitative estimation were also poorly efficient, mainly based on gravimetric and volumetric techniques [106]. The analysis of large amounts of samples was arduous, time-consuming, and required considerable expertise from the analyst. Currently, due to the development of new analytical instruments and procedures, it is possible, through so-called tandem or hybrid techniques, to separate the desired components from a matrix and, coupled with the separation instrument, identify and quantify them through spectroscopic devices.

While many different methods have been explored over the last years in terms of analytical tools to identify and quantify compounds in cork by-products, the most commonly chosen for this purpose have been gas chromatography coupled with mass spectrometry (GC-MS), HPLC coupled with diode array detection (HPLC-DAD) and the Folin-Ciocalteu method.

Gas chromatography (GC) is one of the most commonly employed techniques in analytical chemistry for the separation, identification and quantification of compounds in a complex mixture [107]. As with all analytical methods, the choice of the detector, according to the desired goal and the sample components, is a central one. A common detector applied to analysis of substances from cork by-products is the mass spectrometer (MS), and the tandem method resulting from its conjugation with gas chromatography (GC-MS) is a popular one for the analysis of components present in cork by-product extracts [57,82], not only for the qualitative identification of compounds, such as triterpenoids and fatty acids, but also their quantitative evaluation. The main limitation associated with this technique is the fact that the sample must be volatile below 300 °C, which makes this technique unsuitable for the analysis of non-volatile materials. A technique which can be used for the analysis of non-volatile or thermally unstable species is liquid chromatography (LC). LC is an appropriate technique for the separation of all sorts of components in a solution, and can be used for analytical, as well as preparative purposes. As with other techniques, the successful utilization of HPLC demands a clear comprehension of how the separation of the compounds is affected by experimental conditions, such as the temperature, flow rate and the choice of column and eluent [108]. Detection can be achieved by ultraviolet-visible detection (HPLC-UV/Vis), mass spectrometry (HPLC-MS), diode array detection (HPLC-DAD), among other. Within solute property detectors, such as the ones mentioned, diode array detectors (DAD) have been widely used for the identification [51,79] and overall analysis [65,98] of cork by-product extract samples. These detectors differ from conventional UVVis detectors in their capacity to monitor an entire spectral range (190–1100 nm) during analysis, whilst being capable of recording spectra simultaneously, which considerably decreases the time of the analysis. Additionally, a DAD can perform peak purity analysis [109]. Diode array detectors, combined with mass spectrometers, are also frequently coupled with LC (LC-DAD-MS), a technique that has been employed by Fernandes et al. [98] for the identification and analysis of compounds present in cork granulates, namely phenolic acids and aldehydes and ellagitannins. Beside DAD, other detectors have been combined with HPLC. Conde et al. [67] used UV detectors for the identification and quantification of phenolics from cork granulates, and Santos et al. [80] used the same technique for the separation of components of extracts from three kinds of cork by-product, cork granulates, black condensate and cork powder. This last author has also resorted to HPLC-MS for the analysis of phenolic components in cork granulate [66].

While MS had been used for the analysis of various kinds of small molecules, it was the development of a “soft desorption ionization” technique using an organic compound matrix that allowed for the analysis of biological macromolecules, such as proteins and oligonucleotides—this method is known as Matrix-Assisted Laser Desorption and Ionization (MALDI). Currently, MALDI is conjugated with Time of Flight (TOF) mass spectrometry for the analysis of various kinds of biomolecules and organic compounds, as well as for clinical applications, such as the identification of microorganisms [110], and diagnosis of pathologies [111]. MALDI is a soft ionization technique, characterized by low fragmentation of molecules and ions, using either UV or infrared (IR) radiation as light source. MALDI-TOF-MS, in particular, is an accurate and sensitive technique, which has been applied, by Reis et al., for the tentative identification of tannins in cork powder [79].

Another method commonly used for identification of chemical species is ^13^C nuclear magnetic resonance (NMR). Nevertheless, the analysis of cork industry by-products by ^13^C NMR is limited to the work performed by Castola et al. [58], who made use of this technique for the identification and quantification of triterpenoids from cork granulates, in accordance with a methodology previously developed by the same authors [112]. This procedure relies upon the computer-aided analysis of the ^13^C NMR spectra of the compounds, without requiring their previous separation from the extract through a conventional chromatographic technique, such as those formerly described. It is an efficient and accurate method, which could be optimized for other families of components in cork, beyond triterpenes.

As cork by-product extracts are generally rich in phenolic compounds, the most frequently used quantification method is the Folin-Ciocalteu assay, also known as the “gallic acid equivalence” assay [51,66,67,80]. This method consists of a colorimetric assay, based on the reaction between phenols/polyphenols and the Folin-Ciocalteu reagent, which results in the formation of a blue-colored product, which can be quantified by visible-light spectrophotometry [113]. Notwithstanding the fact that it allows for the determination of the total phenolic content of a sample, it is not a selective assay, as the Folin-Ciocalteu reagent will react not only with phenols, but with any species capable of reducing it. However, it is a very simple and practical method, with considerable reproducibility, and various approaches have been proposed to increase the specificity and selectivity of the assay [114]. Another colorimetric assay has been employed for the determination of the total flavonoid content in cork granulate samples, a method based on the formation of a yellow flavonoid-aluminum complex, which can be spectrophotometrically quantified using quercetin as standard [57].

## 5. Biological Activity Evaluation

Once the compounds have been extracted, purified and identified, it is of paramount importance to evaluate their biological potential. In cork, particularly, many compounds present in the described by-products are known to have promising antioxidant properties. These properties, together with their potential to act as antiaging, seborregulator, exfoliant and depigmenting agents [21] enhance the applicability of these compounds for the cosmetic industry—as such, antioxidant capacity is the most evaluated pharmacological property of the extracts. However, evaluation of antimicrobial and antitumor activity for cork extracts has also been reported.

### 5.1. Antioxidant Capacity

The most commonly used antioxidant assay for plant extracts is the 2,2-diphenyl-1-picrylhydrazyl (DPPH) assay [115]. DPPH is a stable nitrogen radical, due to the delocalization of its spare electron over the molecule; this delocalization is the reason for the purple color that characterizes this compound, which has an absorbance maximum at 515–520 nm. When DPPH comes in contact with a substance capable of donating a hydrogen atom, it suffers reduction, and the reduced form of DPPH no longer exhibits color, due to a significant reduction of absorbance at 515–520 nm. The decolorization is stoichiometric in relation to the number of electrons taken up by the compound in analysis, which makes this a simple, rapid and economic method to evaluate the free radical scavenging ability of compounds in cork extracts [116]. The many works in which this assay has been employed for the measurement of antioxidant capacity in extracts from cork by-products are presented in Table 2, as well as the resulting data, expressed in terms of IC_50_.

Another colorimetric method commonly used for the estimation of total antioxidant capacity of natural products is the 2,2′-azino-bis(3-ethylbenzothiazoline-6-sulfonic acid) (ABTS) assay. The first protocol developed for this assay [117] has been subjected to many alterations, resulting in numerous variations of the assay, having to do with different methods to generate the radical cation, different quantification strategies, and different experimental conditions. However, the general protocol involves the conversion, in solution, of ABTS to its radical cation, which presents blue color and maximum absorption of light at defined wavelength values. The addition of a compound with antioxidant effect to the solution results in the quenching of the absorption, and discoloration of the solution, proportionally to the concentration of the compound with scavenging effect [118]. The ABTS scavenging assay is a good alternative to the DPPH assay, as this radical presents solubility in organic and aqueous media, over a wide range of pH values and allows for the evaluation of the antioxidant capacity of both lipophilic and water-soluble compounds [119]. This method has been applied to cork by-product extracts, more specifically to measure the antioxidant activity of compounds present in cork granulate extracts [57] (Table 2).

Alternatively, the ferric-reducing ability of plasma (FRAP) assay, sometimes designated as ferric-reducing antioxidant power assay, also provides a sensitive and rapid approach for the estimation of the antioxidant capacity of an extract [120]. While the two previous methods measure antioxidant power in terms of radical scavenging capacity, the FRAP assay measures antioxidant power on the basis of reducing ability. A solution containing ferric ion (FeIII) complexed with tripyridyltriazine (TPTZ) will suffer reduction to the ferrous form (FeII-TPTZ) when in contact with an antioxidant species; the FeII-TPTZ complex presents blue color and maximum absorption at 593 nm. Due to the variation of absorption being linear over a wide range of antioxidant concentrations, it can be monitored to estimate the reducing capacity and, therefore, antioxidant power, of a sample. This method, however, is not ideal, as the reduction reaction is non-specific, that is, if the half-reaction consisting of the oxidant of the antioxidant species has a less-positive redox potential than the half-reaction consisting in the reduction of the FeIII-TPTZ complex, then this half-reaction will be favored. Additionally, if the sample contains a compound capable of chelating iron, it might interfere with the assay, leading to erroneous low values of antioxidant activity; however, the chances of this occurring are not likely [121]. The application of this method to cork by-product extracts has been reported by Fernandes et al. (2009), as presented in Table 2, to evaluate the reducing capacity of tannins [65].

Additionally, Touati et al. (2015) have also applied an alternative method of measuring reducing power in cork extracts, through the measurement of the conversion of a Fe^3+^/ferricyanide complex to its ferrous form [57]. The results demonstrated that the reducing capacity of the samples (methanol:water cork granulate extracts) was dose-dependent.

As with all in vitro assays, the main drawback associated with these methods (DPPH, ABTS and FRAP assays) is that the experimental conditions do not entirely match physiological ones. Furthermore, some of the reactants used for the assay are not found in nature, which is the case with ABTS—this radical may not be a good model for “physiological” radicals (such as O_2_^•−^, HO^•^ and NO^•^), due to its steric hindrance and large dimensions, which means that the accessibility of the radical plays a part in the reaction. Some of these advantages might limit the ability of these assays to accurately estimate the antioxidant capacity of a sample in a physiological context. Nevertheless, these assays allow a good comprehension of numerous chemical mechanisms, generating comparable and reproducible results without requiring elevated costs or highly specialized technicians.

### 5.2. Antimicrobial Activity

The ever-increasing prevalence of drug-resistant and even multidrug-resistant bacteria represents one of the greatest challenges to health systems all over the globe. This threat has led to an aggravated need to search for new compounds, capable of inhibiting bacterial growth and/or proliferation. Alternative sources of compounds are necessary, and, within this context, the antimicrobial properties of plant extracts have been recognized for many years. As stated above, many substances present in cork and cork by-products present antimicrobial activity, such as the ones included in the phenolic fraction, but also tannins and suberin fatty acids. Therefore, the screening of antimicrobial action within cork by-product extracts is an interesting one, albeit not common.

Touati et al. (2015) evaluated the antibacterial activity of a methanol:water extract of cork granulates, constituted by a variety of phenolic compounds, tannins and others, through a diffusion test in Mueller-Hinton agar, using gallic and tannic acids as positive controls [57]. In this type of method (disk diffusion tests), the activity is expressed as the diameter of growth inhibition. It is used mostly for initial screenings of activity, and to evaluate the range of activity against different strains [122], which was the aim of the case of the mentioned work. Of the four strains tested, two of which Gram-positive and two Gram-negative, the cork granulate extract was effective against *Staphylococcus aureus* and *Pseudomonas aeruginosa*, but not against *Escherichia coli* nor *Listeria innocua*. A more suitable method for the obtention of more quantitative results, despite being more time-consuming, consists in the determination of the minimum concentration at which a compound inhibits bacterial growth (MIC) and the minimum bactericidal concentration (MBC) through the dilution method in agar or broth. None of the works referred to in Table 2 have determined these values for the compounds present in the cork by-product extracts tested.

Neither of these methods, however, provide an indication of the mode of action of the antimicrobial agent. The comprehension of the underlying mechanisms of the antimicrobial activity of plant extracts, and cork by-products in particular, is essential and must constitute one of the next steps in the exploitation of their components as potential antimicrobial agents.

### 5.3. Other Assays

In addition to the biological activity assays described above, an assay uncommonly performed on cork extracts, but useful nevertheless—in particular, because many compounds identified in cork extracts have exhibited antitumor activity against different kinds of cancer—is the Sulforhodamine B (SRB) assay, one of the most used methods for in vitro cytotoxicity screening. This procedure is based upon the stoichiometric binding of SBR (a pink dye) to protein components of cells, which allows for the determination of cell mass, as it is proportional to the amount of dye extracted from the cells once they have been stained [123]. The SRB assay was performed by Fernandes et al. [65] to assess the effect of tannins from cork granulate (described in Table 2) on the proliferation of human tumor cell lines (MCF-7, Caco-2, HT-29). Overall, the entirety of the tested compounds exhibited dose-dependent growth inhibition. This cytotoxicity should be further studied and explored, as the potential medicinal applications are numerous, and due to the authors’ report that the most cytotoxic and effective antiproliferative compounds were also antioxidant agents.

The importance of bioassays is unquestionable, as these complement the chemical and physical characterization of compounds and provide valuable knowledge on their biological activity, as well as potency. This information is critical for industries which could be interested in the valorization of cork by-products. All information regarding extraction, purification, identification, and quantification of compounds in cork by-products, as well as biological assays, was compiled in Table 2.

## 6. Conclusions

Plants are a rich source of bioactive compounds, attractive to many industries—the cork oak is no exception. Its main purpose and application (the production of cork stoppers for the wine market) generate a myriad of residues, which are potential sources of interesting compounds, with many bioactive properties, ranging from antimicrobial activity, to antioxidant, anti-inflammatory, antiproliferative, among others. These distinct bioactivities are attractive to the cosmetic and pharmaceutical industries. The cosmetic industry, in particular, could find new applications for these residues, especially taking into account the wide array of promising properties they have exhibited, such as antioxidant, depigmenting, seborregulator, exfoliant, and antiaging [21,124]. A relevant example due to its high market demand is the antiaging potential of the cork extracts: several compounds found in cork extracts have demonstrated the ability to inhibit enzymes such as collagenase, elastase [125,126], hyaluronase and tyrosinase [127,128], all of which play a key role in skin aging. However, it is not clear whether these compounds retain such biological activities when isolated from cork by-products, or if that ability results from synergistic effects. Other bioactivities, which are relevant to fight the skin aging process, such as the promotion of the synthesis of collagen and glycosaminoglycans, have also been reported for some of cork extracts’ constituents [129,130].

This potential, coupled with both the increasing concern for environmental protection and the growing awareness from consumers about the sources of cosmetic ingredients, has made cork by-products a promising sustainable source of bioactive ingredients. In turn, this has led to a wider demand for more efficient extraction and analysis techniques for these compounds. The current work has focused on compiling these techniques, previously described in the literature, to extract and characterize cork industry by-products, in order to incentive the exploitation of available technologies towards the enhancement of the use of these by-products as sources of bioactive compounds, with pharmaceutical and/or cosmetic utility. A wide array of methodologies has been described, including conventional and modern extraction techniques, with a clear predominance of techniques that resort to environmentally harmful solvents and high consumptions of energy. The descriptions of the use of greener techniques are still scarce and need to be better exploited. Regarding analytical methods for identification and quantification, classical GC and LC methods were described, but the use of two-dimensional liquid chromatography (2D-LC) or GC × GC, strategies already in use in the natural products field, were not reported.

While many studies have been carried out regarding the composition of cork by-products and waste residues, some are still insufficiently characterized, as is the case of black condensate and cork wastewater. In order to fully assess the potential of these by-products, further studies are required, as well as new strategies to reduce their environmental impact, specifically concerning boiling wastewater. Additionally, the reports on toxicological data for the compounds present in these and other by-products are scarce, and would be a powerful tool, alongside in vivo compatibility assays to confirm their application in health products. The pharmaceutical and cosmetic potential of the residues generated by the cork industry is vast, and future efforts should be deployed for a better comprehension of their biological effects, as well as developing approaches to exploit them safely and sustainably.

## Figures and Tables

**Table 1:** Common compounds found in different cork by-products, based on the yields obtained from extractions described in literature.

Cork By-Products			
Granulates	Agglomerates	Powder	Cooking Wastewaters
**Ellagic acid (17)**	Friedelin (**4**)	Betulinic acid (**7**)	Ellagic acid (**17**)
**Gallic acid (12)**	β-sitosterol (**10**)	Cerin (**5**)	Gallic acid (**12**)
**Protocatechuic acid (13)**	Betulin (**6**)	Friedelin (**4**)	Protocatechuic acid (**13**)
**Cerin (5)**	Betulinic acid (**7**)	Ellagic acid (**17**)	Ferulic acid (**16**)
**Friedelin (4)**	Ferulic acid (**16**)	Betulin (**6**)	Vanillic acid (**14**)
**Betulinic acid (7)**	Coniferyl aldehyde (**20**)	Castalagin (**24**)	Syringic acid
**Betulin (6)**	Aesculetin (**22**)	Tergallic-c-Glucose	

**Table 2:** Different procedures for the extraction, purification, identification, and quantification of compounds in cork by-products, as well as biological assays commonly employed for their characterization.

By-Product Compound(s)	Yield (mg of Compounds/kg Dry Cork By-Product)	Sample Preparation Extraction and Purification	Identification and Quantification	Biological Assays	Ref.
**Cork granulate** **Ellagic acid (17)** **Protocatechuic acid (13)** **Vanillic acid (14)** **Gallic acid (12)** **Vanillin (19)** **Scopoletin (23)** **Caffeic acid (15)** **Coniferaldehyde (20)** **Ferulic acid (16)** **Protocatechuic aldehyde (18)** **Aesculetin (22)** **Sinapaldehyde (21)**	228.4 48.8 27.4 18.3 16.1 12.7 12.1 11.2 10.7 8.1 7.5 4.5	Grinding and sieving of cork material; Extraction with MeOH:H_2_O (80:20, *v*/*v*), for 24 h, at room temperature; Filtration of the suspension, removal of MeOH by vacuum distillation; Aqueous solution extracted with Et_2_O.	HPLC-UV (Identification) GC-MS Folin-Ciocalteu Method	-	[67]
**Cork powder** **Betulinic acid (7)** **Cerin (5)** **Friedelin (4)** **Betulin (6)** **β-Sitosterol (10)** **Ursolic acid (8)** **Lupeol** **Ellagic acid (17)**	11,719 2060 2009 875 254 104 60 1347	Sequential Soxhlet extraction with DCM, MeOH and H_2_O for 10 h with each solvent.	GC-MS	-	[82]
**Black condensate** **Friedelin (4)** **β-Sitosterol (10)** **Betulin (6)** **Betulinic acid (7)** **Ferulic acid (16)** **Vanilylpropanoic acid** **Benzoic acid**	95,290 20,930 13,130 12,080 10,960 5360 3480	Soxhlet extraction with DCM for 10 h; Alkaline hydrolysis with 0.5 M NaOH; Extraction with DCM 3x.	GC-MS		[82]
**Cork granulate** **Friedelin (4)** **Betulinic acid (7)** **Sitost-4-en-3-one (11)** **Betulin (6)**	21.0 * 7.3 * 2.9 * 2.7 *	Soxhlet extraction with DCM for 8 h.	^13^C NMR (identification and quantification)		[58]
**Cork granulate** **Friedelin (4)** **Sitost-4-en-3-one (11)** **β** **-Sitosterol (10)** **Betulinic acid (7)** **Betulin (6)**	20.4 * 15.0 * 2.9 * 2.1 * 1.4 *	Supercritical CO_2_ extraction at 50 °C and 220 bar for 6 h.	^13^C NMR (identification and quantification)		[58]
**Cork granulate** **Castalagin (24)** **Tergallic-C-Glc** **Roburin D** **Vescalagin (25)** **Vescavaloninic acid (32)** **Roburin A (28)** **Roburin E (27)** **Castavaloninic acid (33)** **Grandinin (26)**	424 264 149 130 118 103 77 67 58	Steam treatment of the cork granulates for removal of volatile compounds; Defatting with chloroform in a Soxhlet system for 8 h; Maceration of cork granulates with an aqueous solution of acetone (90%), for 150 h at 25 °C; Precipitation of supernatant with 4 volumes of ethanol (96%); Centrifugation at 10,000 rpm for 10 min at 4 °C; Liquid-liquid extraction with ethyl acetate; Purification of aqueous phase by solid-phase extraction; Elution of tannins fraction with a mixture of methanol/acetone/water (3:1:1, *v*/*v*/*v*).	HPLC-DAD MALDI-TOF		[79]
**Cork powder** **Castalagin (24)** **Tergallic-C-Glc** **Vescalagin (25)** **Roburin D** **Roburin A (28)** **Vescavaloninic acid (32)** **Grandinin (26)** **Roburin E (27)** **Castavaloninic acid (33)**	842 440 346 324 258 257 193 190 185	Defatting with chloroform in a Soxhlet system for 8 h; Maceration of cork powder with an aqueous solution of acetone (60%), for 9 h 30 min at room temperature; Precipitation of supernatant with 4 volumes of ethanol (96%); Centrifugation at 10,000 rpm for 10 min at 4 °C; Liquid-liquid extraction with ethyl acetate; Purification of aqueous phase by solid-phase extraction; Elution of tannins with a mixture of methanol/acetone/water (3:1:1, *v*/*v*/*v*).	HPLC-DAD MALDI-TOF		[79]
**Cork granulate** **Di-HHDP-glc** **HHDP-di-galloyl-glc** **Di-HHDP-galloyl-glc** **HHDP-tri-galloyl-glc** **Castalagin (24)** **Mongolicain B**	NA	Grinding and sieving of cork to obtain 0.5–1 mm particles; Extraction with 12% ethanol, 5 g/L tartaric acid (pH 3.2) for 72 h at room temperature (wine-model solution); Filtration by gravity, removal of the ethanol by vacuum distillation; Spray drying of the aqueous residue, and extraction of the obtained powder with ethyl acetate 3 times; Isolation and purification of phenolic compounds by preparative HPLC, with two solvents: (1) H_2_O/CH_3_COOH (92:2, *v*/*v*) and (2) CH_3_COOH/CH_3_CN/H_2_O (2:20:78, *v*/*v*/*v*)	HPLC-DAD LC-DAD/ESI-MS	DPPH scavenging assay (Avg ~25 μM equiv. Trolox); FRAP method; SRB Assay (Avg ~20 μM equiv. Trolox).	[65]
**Cork granulate** **Protocatechuic aldehyde (18)** **Vanillin (19)** **Protocatechuic acid (13)** **Gallic acid (12)** **Coniferaldehyde (20)** **Caffeic acid (15)** **Ferulic acid (16)** **Ellagic acid (17)** **Ellagic acid-pentose** **Ellagic-acid deoxyhexose** **Ellagic acid-hexose** **Valoneic acid dilactone** **HHDP-glc** **Valoneic acid** **Tergallic-C-glucoside** **HHDP-galloyl-glc** **Trigalloyl-glc** **Di-HHDP-glc** **HHDP-digalloyl-glc** **Tetragalloyl-glc** **Castalagin/Vescalagin (24/25)** **Trigalloyl-HHDP-glc** **Mongolicain A/B**	NA	Grinding and sieving of cork to obtain 0.5–1 mm particles; Extraction with 12% ethanol, 5 g/L tartaric acid (pH 3.2) for 72 h at room temperature (wine-model solution); Filtration on a Büchner funnel, removal of the ethanol by vacuum distillation; Spray drying of the aqueous residue, and extraction of the obtained powder with ethyl acetate 3 times; Fractionation of phenolic compounds by column chromatography, using MeOH as eluent.	HPLC-DAD LC-DAD/ESI-MS		[98]
**Cork granulate** **Ellagic acid (17)** **Gallic acid (12)** **Protocatechuic acid (13)** **Caffeic acid (15)** **Aesculetin (22)** **Vanillin (19)** **Vanillic acid (14)** **Ferulic acid (16)** **Coumaric acid** **Salicylic acid** **Eriodictyol** **Naringenin**	2031.5 30.6 17.5 57.6 4.9 14.3 Trace Trace Trace 32.7 27.4 2.6	Milling of the cork planks to obtain cork granulate; Soxhlet extraction with DCM for 6 h; Suspension in MeOH:H_2_O (80:20, *v*/*v*) at room temperature, for 24 h; Filtration and removal of the solvent by low-pressure evaporation; Extraction with diethyl ether 3 times.	HPLC-MS Folin-Ciocalteu method	DPPH scavenging assay (IC_50_ = 2.79 ± 0.15 μg of extract/mL)	[66]
**Cork granulate** **Ellagic acid (17)** **Gallic acid (12)** **Protocatechuic acid (13)** **Caffeic acid (15)** **p-Hydroxybenzoic acid**	526.5 241.6 118.3 12.9 1.0	Milling of the cork planks to obtain cork granulate; Soxhlet extraction with DCM for 6 h; Extraction with MeOH for 6 h; Removal of solvent by low-pressure evaporation; Reflux with water for 6 h.			
**Cork granulate** **Gallic acid (12)** **Ferulic acid (16)** **Caffeic acid (15)**	61.2 7.5 6.5	Semi-continuous subcritical water extraction, with a pressure of 100 bar and temperature range of 50–120 °C.	HPLC-DAD Folin-Ciocalteu method	DPPH scavenging assay (EC_50_ = 0.253 ± 0.001 mg extract/mg DPPH)	[51]
**Cork granulate** **Ellagic acid (17)** **Ellagic acid-pentoside** **Gallic acid (12)** **Aesculetin (22)** **Quinic acid** **Methyl gallate** **Brevifolin-carboxylic acid** **Protocatechuic acid (13)** **Ferulic acid (16)** **Coniferyl aldehyde (20)** **p-Hydroxyphenyllactic acid** **Valoneic acid dilactone** **Caffeic acid isoprenyl ester** **Isorhamnetin-rhamnoside** **Eriodictyol** **Isorhamnetin**	1246.46 770.16 736.48 391.59 372.86 251.43 102.03 79.26 Trace Trace Trace 168.01 127.98 Trace Trace Trace	Soxhlet extraction with DCM for 6 h; Extraction with MeOH:H_2_O (50:50, *v*/*v*), for 24 h, at room temperature.	HPLC-UV (separation) ESI-MS (analysis) Folin-Ciocalteu	DPPH scavenging assay (IC_50_ = 4.77 ± 0.02 μg of extract/mL)	[80]
**Black condensate** **Coniferyl aldehyde (20)** **Aesculetin (22)** **Gallic acid (12)** **Quinic acid** **Ellagic acid (17)** **p-Hydroxyphenyllactic acid** **p-Coumaric acid** **Vanillin (19)** **Caffeic acid (15)** **Protocatechuic acid (13)** **Ferulic acid (16)** **Eriodictyol**	194.34 125.28 118.46 117.17 52.52 49.36 35.76 32.47 17.68 9.97 Trace Trace	Soxhlet extraction with DCM for 6 h; Extraction with MeOH:H_2_O (50:50, *v*/*v*), for 24 h, at room temperature.	HPLC-UV (separation) ESI-MS (analysis) Folin-Ciocalteu	DPPH scavenging assay (IC_50_ = 1.57 ± 0.01 μg of extract/mL)	[80]
**Cork powder** **Ellagic acid (17)** **Gallic acid (12)** **Aesculetin (22)** **Quinic acid** **Methyl gallate** **Ellagic acid-pentoside** **Valoneic acid dilactone** **Protocatechuic acid (13)** **Ferulic acid (16)** **Coniferyl aldehyde (20)** **Caffeic acid isoprenyl ester** **Brevifolin-carboxylic acid** **Isorhamnetin-rhamnoside** **Isorhamnetin**	527.59 263.04 176.80 137.02 96.93 46.18 46.05 16.44 14.77 Trace 82.47 53.72 Trace Trace	Soxhlet extraction with DCM for 6 h; Extraction with MeOH:H_2_O (50:50, *v*/*v*), for 24 h, at room temperature.	HPLC-UV (separation) ESI-MS (analysis) Folin-Ciocalteu	DPPH scavenging assay (IC_50_ = 3.33 ± 0.02 μg of extract/mL)	[80]
**Cork granulate** **Cerine (5)** **Friedelin (4)** **Betulinic acid (7)** **Betulin (6)** **Sitost-4-en-3-one (11)** **β** **-Sitosterol (10)** **Ursolic acid (8)** **Docosanoic acid** **Hexadecanoic acid** **Octadecenoic acid** **Tetracosanoic acid** **Cis-9-octadecenoic acid** **9,12-Octadecadienoic acid** **Nonanedioic acid** **Docosan-1-ol** **Tetracosan-1-ol** **Eicosan-1-ol**	7278.7 4702.3 2798.2 1651.2 1175.2 514.6 435.7 338.9 294.6 181.3 160.2 149.1 129.2 125.5 1409.4 412.0 295.9	Soxhlet extraction with DCM for 6 h; Alkaline hydrolysis with 0.5 M NaOH; Extraction with DCM 3 times.	GC-MS	-	[57]
**Cork granulate** **Protocatechuic acid (13)** **Ellagic acid (17)** **Gallic acid (12)** **HHDP-glc** **Castalagin/vescalagin (24/25)** **Methyl gallate** **Brevifolin-carboxylic acid** **Syringaldehyde** **Valoneic acid dilactone** **Caffeic acid (15)** **Ellagic acid-pentoside** **Chlorogenic acid** **Vanillic acid (14)** **Caffeic acid isoprenyl ester** **Ellagic acid-rhamnoside** **Vanillin (19)** **Isorhamnetin-rhamnoside**	1414.41 1060.47 931.01 678.88 490.89 330.02 231.19 213.92 124.34 112.09 78.82 69.45 67.74 64.89 60.06 42.30 26.18	The residues obtained after DCM extraction (described above) were suspended in MeOH:H_2_O (50:50, *v*/*v*) for 24 h, at room temperature.	UHPLC-MS	DPPH assay (IC_50_ = 5.60 ± 0.020 μg of extract/mL); ABTS assay (IC_50_ = 27.55 ± 0.70 μg of extract/mL); Reducing power (dose-dependent); Mueller-Hinton agar diffusion test	[57]

* Values expressed as mass percentage (measured by ^13^C NMR).
